# Analysis of Bio-Obtainable Endocrine Disrupting Metals in River Water and Sediment, Sewage Influent/Effluent, Sludge, Leachate, and Concentrated Leachate, in the Irish Midlands Shannon Catchment

**DOI:** 10.1155/2009/325496

**Published:** 2010-02-03

**Authors:** Antoinette M. Reid, Concepta A. Brougham, Andrew M. Fogarty, James J. Roche

**Affiliations:** Endocrine Disruption Group, Department of Life and Physical Sciences, School of Science, Athlone Institute of Technology, Dublin Road, Athlone, Co. Westmeath, Ireland

## Abstract

The application of an acid digestion and subsequent solid-phase extraction (SPE) procedure were implemented as preliminary treatments prior to quantifying the levels of potentially endocrine disrupting metals (EDMs) in a variety of solid and liquid matrices. These included (solid) river sediment, leachate sediment and sewage sludge and also (liquid) river water, landfill leachate, concentrated leachate, sewage influent, and sewage effluent, sampled in the Irish Midlands. The total concentrations of cobalt (Co), cadmium (Cd), copper (Cu), chromium (Cr), nickel (Ni), lead (Pb), zinc (Zn), and manganese (Mn), after extraction and preconcentration, were determined by atomic absorption spectroscopy (AAS). Mercury (Hg) in sediment and sludge was determined using cold-vapour atomic fluorescence spectroscopy (AFS). For sewage sludge maximum values (mg/kg_dw_) of 4700 Ni, 1642 Mn, 100.0 Cd, 3400 Zn, 36.70 Co, 750.0 Pb, 485.8 Cr, and 1003 Cu were determined whilst in leachate sediment, maximum values (mg/kg_dw_) of 32.10 Ni, 815.0 Mn, 32.78 Cd, 230.3 Zn, 26.73 Co, 3525 Pb, 124.9 Cr, and 50.13 Cu were found. Over several months, the data showed elevated levels in sewage influents, effluents, and sludges compared to a battery of adjacent river water samples and corresponding sediments. There was a definite trend for target values for sediments to be exceeded, while intervention values were only exceeded for cadmium. Overall the pattern in terms of concentration was sewage > leachate > river matrices. A nonparametric assessment of the effect of sewage treatment method on median metal levels in sludge revealed statistically significant differences at the 95% level of confidence for Co, Cr, and Hg and at the 90% level of confidence for Cd.

## 1. Introduction

Arising from hydro-geological interactions within riverine systems, elements and metals coexist in relative proportions to each other and these ratios are dependent on geochemical processes such as weathering, atmospheric deposition, rainfall and flow-rates. Overall, this process occurs from a dynamic equilibrium between outside exposure from the environment and uptake, storage, excretion, or degradation within the organism. Where elevated levels of metals occur, bioaccumulation can arise, which may be described as an increase in concentration of a chemical over time in a biological organism compared to the chemical's concentration in the environment. There are three main groups of toxic metals that may bioaccumulate. The first group includes lead, cadmium, and mercury, which are toxic at all concentrations and have no known biological functions. With medium toxicity and no known biological function, is antimony (not reported here) and the third group includes copper, zinc, and cobalt, which are essential for various biochemical and physiological processes and are also toxic above certain concentrations. The toxicity will also depend on the oxidation state; for example, Cr (III) is required by the body in trace amounts whilst Cr (VI) is highly toxic. This study looks at the levels of those metals suggested by Kime [1], amongst other commentators, as being potentially oestrogenic in matrices subject to extensive human inputs in the Irish Midlands.

The most important point to note about EDMs is that they are not biodegradable, therefore their monitoring and quantitation in the environment is indicated. When metals are discharged into the aquatic environment, they tend to partition, as part of a process known as speciation, into sediment and form chemical fractions, which reduces the availability of the free hydrated metal ion. According to Korfali and Davies [2], the interaction between metals and carbonates is important as the carbonates provide a means of scavenging metals such as Zn, Pb, and Cd. They demonstrated that river sediment in areas of limestone had a self-purification capacity for metal pollution due to associations of these metals with carbonates. Pb and Cd in particular were found to have a high association with the carbonate fraction and to a lesser extent with iron oxide/hydroxide fractions. Carbonates are also known to neutralise a variety of chemical wastes and the elements may become incorporated into the calcite lattice, hence immobilising them. Also in areas of limestone, such as the Shannon Catchment in the Irish Midlands, the river will have a slightly alkaline pH, which lowers desorption of metals and has a buffering capacity against any acidic inputs or discharges. The chemical form of the metal will determine the mobility in aquatic media, and metals may act as pollutants if significant changes in pH, redox potential, salinity, particulate matter or microbial activity occur. In this study, analysis was carried out for Cu, Co, Cr, Ni, Mn, Zn, Cd, and Pb via AAS whilst Hg in sediment was measured using cold-vapour AFS (Chemex, UK). Thus far, most research into compounds suspected of being capable of inducing hormone-modulating effects has focussed on organic molecules and consequently inorganic species possessing similar capabilities have been somewhat overlooked. The objectives of this study were to quantify the levels and ranges of EDMs and to provide relevant, baseline Irish values for those matrices typically studied by toxicologists seeking to determine and validate oestrogenic effects based on EC_50_ values.

## 2. Materials and Methods

### 2.1. Reagents and Standards

All reagents were of analytical grade. Metal reference standards purchased from Reagecon (Ireland) were diluted in 3 M HNO_3_ (69% HNO_3 _AnalaR BDH, Sigma-Aldrich; diluted with ultrapure deionised water) on a daily basis to give 0.5, 1.0, 1.5, 2.0, 2.5, and 3.0 *μ*g/mL standard solutions. 47 mm microfibre glass GF/C (1.2 *μ*m), GF/F (0.7 *μ*m), and 0.45 *μ*m nylon filters were obtained from AGB Scientific, Ireland. 3M-Empore metal chelating resin SPE disks were purchased from JVA Analytical Ltd (Ireland). 

### 2.2. Rationale for Sampling Handling and Choice of Extraction Vehicle

Both solid and liquid samples were taken at repeat sampling locations using an inert, stainless steel, telescopic sampling rod and cup followed by transfer to a precleaned acid washed, 2.5 L bottle (liquid) or a sample jar (solid), with a regime for the latter of selecting the upper 2.5 cm–7.5 cm of sediment. Samples were then stored in cooler bags and transported to the laboratory directly, following physical characterisation. Sediment samples were dried to constant weight and stored at 4°C for analysis at a later time; whilst liquid samples were treated with 2 mL of 5 M hydrochloric acid (in order to ensure that the elements were present in only one oxidation state and suitably preserved) and filtered to 0.45 *μ*m before extraction by SPE. Solid samples of particle size <65 *μ*m were finely ground for analysis using an acid washed pestle and mortar and the fine sediment fraction was then weighed out. Given the context of this study, the variety of matrix types and their composition, it was decided based on preliminary studies that 3M HNO_3_ was the best extraction vehicle for both liquid and solid sample protocols. Also, dilute nitric acid is known to possess the ability to dissolve almost all elements that could become environmentally available. Tipping et al. [5] performed pseudototal metal extraction using 0.43 M HNO_3_ whilst Sabienë and Brazauskienë [6] demonstrated that 2 M HNO_3_ extracted analogous amounts of heavy metals to *aqua regia*. Lee et al. [7] examined nitric acid concentrations ranging from 0.02 to 0.10 N for studying the kinetics of heavy metal extraction for sludge particles of 212 *μ*m and found that metal extraction rates increased with acid concentration and temperature but decreased with increasing particle size. The pseudototal metal determination is more representative of what can become bioavailable as opposed to performing a total metal content determination through performing sequential fractionation of samples [6]. For the comprehensive analysis of metals, nitric acid will extract from exchangeable, carbonate, iron/manganese oxide, and organic matter/sulphide fractions and to a lesser extent from the residual fraction [8]. Similarly, Song and Greenway [9] preferred usage of nitric acid for digestion of composts as opposed to *aqua regia* which is recommended in ISO 11466 for total digestion of soil and sediment samples. Hseu [10] also indicated nitric acid as the most efficient extraction solvent for metals based on recovery performances of metals in composts, in which comparisons between dry ashing along with digestions using nitric, nitric-perchloric acid mixes, and sulphuric acid methods were investigated.

### 2.3. Sampling and Preliminary Sample Clarification

Water samples were prefiltered with a 0.45 *μ*m nylon filter to remove particulate matter, which could block sorbent pores. For the analysis of liquid matrices, analytes from 500.0 mL of river water, 200.0 mL of influent, and 500.0 mL of effluent, respectively, were extracted using SPE disks incorporating a metal chelating resin. All final extracts were diluted to 25.0 mL after collection and calculations adjusted accordingly. 

**Figure 1:**
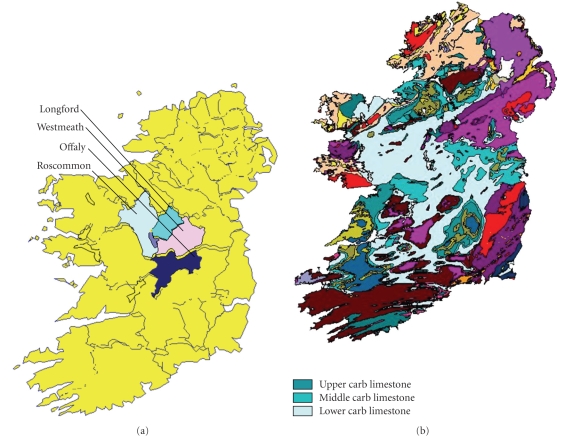
The test areas and the geological attributes of these areas (Ordnance Survey Ireland/Government of Ireland: Permit No. MP008109).

**Figure 2:**
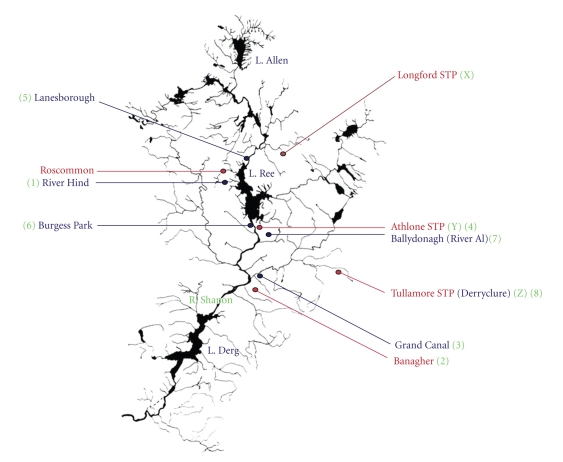
The Shannon Catchment test locations.

Several river sites along the river Shannon, including three of its tributaries, three sewage treatment plant types and three landfill types were selected for the analysis of sediment, sludge, and leachate sediment, respectively, from the counties Longford, Westmeath, Roscommon, and Offaly (Figures 1 and 2). 

### 2.4. Extraction of Liquid and Leachate Samples

For solid-phase extraction of the liquid samples, a three-station vent discharge filtration manifold was assembled. The disk consists of a cross-linked polystyrene-divinylbenzene polymer support that is functionalised by bonding at the nitrogen atoms to imino-diacetate groups which possess the ability to selectively extract and preconcentrate multivalent metal cations. The charge on the functional group will depend on the pH of the solution; at pH 2 the carboxylate groups will be neutral, but the nitrogen will have an overall positive charge and the molecule will have a weak anion exchange capability. As the pH approaches 7 the molecule functions as a cation exchanger (chelator). The chelator binds better with alkaline earth metals than with alkali metals. The disk capacity is quite high. A matrix pH of ≥5 is preferred and elution of extracted metals may be carried out using 3 M HNO_3_ or 3 M HCL. The use of these disks has recently been comprehensively reviewed elsewhere [11].

Preswelling of the polymer was followed by addition of 20 mL of 3 M HNO_3_ to the reservoir with a small amount pulled through initially under tap vacuum, but allowing the disc to soak for about 1 minute. The remaining solvent was eluted and the disc allowed dry. Conditioning with a solvent was not required as the disk was already a hydrophilic sorbent. Two separate 50 mL portions of ultra-pure water were used to wash away traces of HNO_3_. Because the chelator exists as a sodium salt of iminodiacetate, it was necessary to initiate its conversion to the ammonium form, which is more active. To do this, 20 mL of 2 M ammonium hydroxide were added. Extraction of sample was then carried out. Elution required two 10 mL aliquots of 3 M HNO_3_ and the final eluate was then diluted to 25 mL using 3 M HNO_3_ as the diluent.

### 2.5. Extraction of Solid Samples

Solid samples were oven-dried to constant weight at 80°C for 24 hours, homogenised using a mortar and pestle, and stored. The organic carbon content, using 2.5 g sediment, was estimated using a standard procedure suggested by Jain [12]. For the determination of pH, 10.0 g of dried sediment were agitated for 10 minutes in deionised water and then allowed to settle for 1 hour before obtaining a measurement. For the carbonate content, 5.00 g of sediment sample was reacted with 1.0 M HCl, with the residual acid being titrated against 1.0 M NaOH. For metal extraction, samples were treated with 3.0 M HNO_3_. Approximately 2.0 g of sediment (<65 *μ*m) was analysed whilst only 1.0 g sludge was used (sample swelled on contact with liquid) and a volume of 40 mL of the selected extracting solvent was then pipetted into the conical flask containing the sample, which was then agitated for 10 minutes using a stir-bar. Agitation was carried out periodically over 48 hours at room temperature to ensure sufficient extraction. The sample was then filtered completely through a Whatman 541 filter paper with the filtrate being collected and transferred to a 50 mL volumetric flask before dilution with 3 M HNO_3_. The metal standards were then prepared in a solvent identical to the sample matrix and all samples were analysed by AAS. 

### 2.6. Quantitation by AAS

Specific lamps for Co, Cd, Cu, Cr, Ni, Pb, Zn, and Mn were used at respective optimum wavelengths of 240.7, 228.8, 324.8, 357.9, 232.0, 217.0, 213.8, and 279.6 nm using a Varian SpectrAA-600 AAS. Calibration was by multiple external standards. Any curvature of the calibration response was accommodated through the use of a new rational calibration algorithm. The AASpectra software facilitates suitable preread delays and averaging of multiple absorption readings. Observed values were adjusted for recovery percentages noted during validation of the method.

### 2.7. Quantitation by Cold Vapour Atomic Fluorescence Spectroscopy (CV-AFS)

A number of samples were selected and supplied to Chemex, UK due to nonavailability of a CV-AFS apparatus in AIT. The samples were dried to constant weight and 10.0 g of sample was weighed out into a suitable plastic container (a blank container was also examined with these samples). The method detection limits and quantitation limits for mercury in these solid samples were 0.05 mg/kg (dry) and 0.25 mg/kg (dry), respectively. These values are not unadjacent to the frequently quoted target value and intervention values of 0.3 and 10 mg/kg, respectively, for Hg, as soil quality objectives in Denmark and the Netherlands (Bak and Jensen [3], and see also Table 1). Chemex then exposed the samples to acid digestion prior to CV-AFS.

## 3. Results and Discussion

The oestrogen receptor is branded as a metal binding protein, with the most notable example being zinc which binds to cysteines in the DNA binding area of the oestrogen receptor (ER) creating a “Zinc finger.” Zn, Ni, and Co may also interact with the hormone-binding domain of the ER. Using an oestrogen receptor transcriptional assay and an oestrogen screen (E-screen) assay, Choe et al. [13] found high oestrogenicity for Cd, followed by Co, Pb, Cr, and Cu, confirming similar work [14], hence introducing metals and metalloids as a new class of nonsteroidal environmental oestrogen. The E-screen uses an MCF-7 human breast cancer cell line for determining oestrogenicity and possesses an oestrogen receptor, which responds in culture to the presence of oestrogens by a proliferation response. Cells are grown in media containing human serum as this contains an inhibitory factor that prevents the cells from proliferating. The presence of natural, synthetic, or other xenooestrogens can override this inhibition resulting in an in vitro method for quantisation of oestrogenic potential. In terms of the endocrine disrupting potential of metals, Martin et al. [14] had found that treatment of MCF-7 cells with metals including Co, Cu, Ni, Pb, Hg, Sn, or Cr, stimulated cell proliferation and, after six days, there was a 2–5 fold increase observed in cell number. In the same study it was found that those metals decreased the concentration of the oestrogen receptor protein and mRNA by 40%–60% and an induced expression of the oestrogen-regulated genes for the progesterone receptor by 1.6–4 fold was observed. Additionally, Choe et al. [13] had found that a range of oestrogenicity was observed for different species of the implicated metals. The EC_50_ may be understood as the effective concentration of a compound or element that produces a 50% alteration in luciferase activity in an in vitro bioassay used to determine oestrogenicity. The relative potency of the following metals was determined as the ratio of the EC_50_ oestradiol (E_2_) to the EC_50_ of the metal in Table 1 [13–15]. Note that the relative potency is often called the oestradiol equivalency factor (EEF) and is determined using the EC_50_ values of E_2_ and the test compound (EC_50_ Oestradiol (E_2_)/EC_50_ Test compound). Similar biological findings based on a battery of in vitro and in vivo assays were observed in (some as yet unpublished) work carried out by other members of the Endocrine Disruption Group at AIT. A separate analysis by the group has been published of levels of tin determined in the sediment matrices [16].

The pH monitoring at our test locations revealed that all were alkaline in nature (Table 2) consistent with the geochemical properties of the area (Figure 1). The organic content (Table 2) ranged from 2.66% to 8.13%. The River Hind (Mote Park, Co. Roscommon), the River Shannon at Burgess Park (Athlone, Co. Westmeath), and the River Al (Ballydonagh, Co. Westmeath) all of which are downstream of landfill, and leachate sediment from Derryclure (Tullamore, Co. Offaly) had metal concentrations consistent also with the theory that higher values may be found in areas where there is a lot of waste or effluent discharging into the watercourse, that is, areas with a high organic residue. Carbonate values for sediments ranged from 19.99% to 24.49%, indicating high buffering capacities of sediment in the Irish Midlands for metal pollution. Unsurprisingly, given their high association for carbonate, both Pb and Cd were repeatedly above target values at all sites. In a report by the Irish Environmental Protection Agency [17], analysis of sediments indicated metal loadings, which were above suggested target values (see Table 3) at locations in the Shannon Catchment for Ni, Cu, Zn, and Cd but were unable to identify the source. For these metals, the report referred to a target and intervention value. If the average contamination concentration in a soil volume of 25 cm^3^ exceeds the intervention value, the contamination is classified as serious. 

In the following data (Table 3) from the locations tested, results were generally, for one set of recommended limits, below intervention values with the exception of Cd. A definite source for this finding was not clear. Two of the four locations were in close proximity to landfill sites.

For sediments tested for Cu, levels between 1 and 100 mg/kg were determined (Table 3), the average of which would compare best with results found by Korfali and Davies [2] (Lebanon) who found levels between 6.65 and 99.5 mg/kg in a range of river sediments tested. For Cr, levels between 9 and 124 mg/kg and Zn 45 and 277 mg/kg were found which compared best with Ramessur and Ramjeawon [18] (Mauritius) who found concentrations of Cr (75–135 mg/kg), and Zn (137–197 mg/kg) in river sediment. Chandra Sekhar et al. [19] (India) found levels (mg/kg) between 356 and 671 for Zn, 205–572 for Cu, and 40–63 for Cr. Our levels for Cr were similar; Zn was lower whilst Cu levels were much lower. In general, the levels determined in Irish sediments were for the most part below intervention levels and metal enrichment of riverbeds did not appear problematic. However, sediment from the Grand Canal (sampling site no. 3), one of two major man-made inland watercourses permeating the Irish countryside (and a busy boating facility) and which feeds into the River Shannon at Banagher, appeared, when sampled over a six-month period, to have higher overall metal concentrations compared with river sediments from the rivers Shannon or Hind. 

In 1999, the level of lead in a mining area located in the south-midlands was between 50 and 3,650 mg/kg_dw_ and further sampling in agricultural areas near this location found levels between 25 and 14,842 mg/kg_dw_ [20]. Soils enriched with Pb were also found to be enriched with Zn. In this study, levels of Pb between 128 and 3,525 mg/kg_dw_ (maximum value) were found. The areas with the highest levels included the old landfill site at Burgess Park and to a lesser extent areas of increased leisure craft activity, namely, at Shannon Harbour and Athlone Lock. Carbonate content was high at all these locations. Similar to the findings at the mining area, soils in the test areas in this study showing elevated levels of lead were also enriched with Zn. The Irish EPA stipulates that dredged sediments in areas of high Pb content (higher than 1,000 mg/kg)_dw_ should not be spread on land where animals can gain access to dredgings. Where certain metals are found to be present in high concentrations, soils should be limed to raise the pH and hence reduce mobilisation of metals in the soil. 

Jensen et al. [21] showed that landfill leachate colloidal fractions and fine sediments (>1.2, 0.4, and 0.001 *μ*m) contained variable but high distribution levels of Cd (38%–45%), Ni (27%–56%), Zn (24%–45%), Cu (86%–95%), and Pb (96%–99%). Similar to Jones et al. [22], they also found that these colloidal bound materials were associated mostly with the organic fraction with the exclusion of Zn, which tends to be associated with inorganic fractions. They demonstrated that Cd, Cu, and Pb are generally associated with dissolved organic carbon, Ni with carbonates, and Zn with carbonates and as free divalent Zn. The main part of the Cu and Pb was in the colloidal fractions. In this study, overall metal levels determined in leachate sediment from Burgess Park were in the following ranges (mg/kg); Zn (81–230); Cd (2–30); Ni (21–31); Cr (11–125); Co (10–27); Cu (22–50); Pb (550–3,525); Hg (0.05–0.12); Mn (31–229), whilst values for Ballydonagh (a lined and managed facility) were; Zn (78–190); Cd (<1–33); Ni (23–32); Cr (10–48); Co (6–21); Cu (7–29); Pb (140–268); Hg (<0.05); and Mn (50–815) (Table 4). For comparison, we sampled an additional landfill (Derryclure, sampling site no. 8) for just one month (April) in the adjacent County of Offaly. Results for this location (mg/kg) were Zn (351); Cd (12); Ni (24); Cr (15); Co (10); Cu (32); Pb (255); Hg (0.2); and Mn (502). These values are lower than reported values for Danish landfills for Zn and Cr, comparable for Ni and Cu, and higher than Cd and Pb determinations [21]. Overall, the old, unlined landfill facility at Burgess Park contained higher levels of Zn, Cr, Cu, Pb, and Hg compared to the other two landfill types. The lined facility at Ballydonagh contained higher levels of Mn than either of the other two whilst Derryclure had the highest overall levels of Zn. 

Overall it may be summarised from the results that the general trends in abundances of the metals for the three landfill types were as follows: Mn > Pb > Zn > Cu > Ni > Cd > Cr > Co. Chandra Sekhar et al. [19] found Zn, Cd, and Cu to be mostly in the mobile fraction (exchangeable and carbonate bound) of sediment and had the greatest bioaccumulation potential in fish, whilst Ni and Co were less mobile and had a lower bioaccumulation potential. In our programme additional testing was carried out on a number of samples of a fixed mass of 10.0 g_dw_ which were subjected to acid digestion, and then analysed by CV-AFS, to quantify the levels of mercury present. The target and intervention values for the Netherlands and Denmark are 0.3 and 10.0 mg/kg [3, 17, 23].

The highest level of Hg in leachate sediment was found at Derryclure Landfill (0.19 mg/kg) from silt taken from an underground trap (Table 5). Burgess Park in Athlone, which is an old unlined landfill facility, had similar levels present with values ranging from 0.05 to 0.12 mg/kg. Comparing these with river sediment locations tested, values were comparatively lower with the exception of Shannon Harbour, which had 0.12 mg/kg detected in the month of January. This location also had elevated levels of other metals which is interesting as this area had a high volume of cruise boats passing through. Otherwise, the results obtained from the sampling of river water were as follows (Tables 6 and 7).

Metals in water from the River Hind and River Shannon (Lanesborough and Athlone Lock) were slightly higher than those at Shannon Harbour or Banagher. Both the Hind and the Athlone Lock tests site are in close proximity to landfill leachate whilst Lanesborough is directly beside a power plant (with associated higher water temperatures). For more extensive analysis of leachate, a number of sites were further examined including concentrated leachate samples from Derryclure Landfill at three down-gradient points on the site. Both ground and surface water from Ballydonagh Landfill and surface water from Burgess Park were also tested. The results from leachate testing are as shown in Tables 8 and 9.

Cd, Ni, Zn, Cu, Cr, and Pb are generally found in leachate at moderate levels in the ranges (2–20 *μ*g/L) Cd, (100–400 *μ*g/L) Ni, (500–2000 *μ*g/L) Zn, (20–100 *μ*g/L) Cu, (100–500 *μ*g/L) Cr, and (50–200 *μ*g/L) Pb as quoted by Jensen and Christensen [24]. Leachate contains various contaminants and varies in composition depending on landfill technology. Concentration levels (*μ*g/L) of metals in leachate as found by the same authors were Cd (0.2–3.6), Ni (28–84), Zn (85–5310), Cu (2–34), Cr (0–188), Pb (0–16). However, colloidal material has a high affinity for metal in leachate, so levels will depend on how much colloidal matter is present in the leachate sample. In this study, the following maximum values ( *μ*g/L) were determined for leachate; Cu (34), Cr (7), Zn (936), Ni (10), Mn (623), and Pb (56). Our determinations compare favourably with those found by Jensen and Christensen [24]. Levels of suspended and dissolved solids were the highest at Derryclure and metals quantified in this leachate correlate accordingly with elevated concentrations principally for Pb, Zn, Ni, and Cu.

Overall, the concentrated leachate from Derryclure had higher levels of Cu, Zn, Ni, Mn, and Pb as expected, whilst there was little difference observed between ground water and surface water from Ballydonagh, indicating that these leachates are not currently adding to metal loadings. Levels of metals, with the exceptions of Pb and Mn, found in both leachate and river waters were well below the recommended limits for drinking water (Table 1). Overall, there were no acute differences between the levels for the three types of landfill. 

The testing of three different types of sewage treatment facilities was carried out and the metal contents of sludges were determined (Table 10). The liquid matrices included sewage influent and sewage effluent (Table 11) and separate analysis was performed on the sludge. The first type of plant was an aerobic facility with secondary treatment only (*X*), the next was aerobic tertiary treatment (*Y*), and the final plant had tertiary anaerobic treatment (*Z*). The levels determined in the sludges were subjected to a nonparametric test for equality of median metal concentration. This Kruskal-Wallis test offers an alternative to the one-way analysis of variance. The relevant hypothesis challenges whether the medians are all equal or are not all equal. The greatest divergence, at the 95% level of confidence, from equality of median was for Cr, with differences also recorded for Co and, albeit with a smaller sample size, Hg. The *P*-value for cadmium was less secure; indeed we determined that the medians were not the same at the 90% level of confidence for Cd. For the other metals, the test informed that we did not have enough evidence to say that the median observations, and hence the treatment types, were not the same.

From this data we can see that the most ubiquitous metals were Ni > Zn > Mn > Pb and this ranking was followed by Cu > Cr which in turn was followed by Co and Cd. 

In terms of the treatment efficiency the overall pattern observed was *Z* > *X* > *Y*, that is, the anaerobic treatment was better (all metals reduced in concentration) than the aerobic secondary, which in turn, somewhat surprisingly and in what possibly appears to be a capacity issue, was better than aerobic tertiary treatment alone. For the latter Zn, Pb, and Cu effluent levels were noted to increase, while decreases were recorded for Ni and Mn. Mn also increased slightly for the aerobic secondary treatment but otherwise this treatment type resulted in falls in metal concentration with treatment. 

On looking at Hg (Table 10), the levels of mercury found in sewage sludge were in the order Tullamore > Athlone > Longford. Therefore, although it was not possible to analyse Hg in our liquid samples, one could reasonably assume that effluent levels would be lower in the same treatment order to the other metals tested. Overall, the highest levels of Hg were found in sewage sludge with the anaerobic system accumulating the most. The overall order was tertiary anaerobic treatment sludge > tertiary aerobic treatment sludge > secondary aerobic treatment sludge. The next highest level was found at Derryclure Landfill and similar levels were found at Burgess Park in Athlone. 

In Athlone STP (*Y*), iron chloride is the main ingredient now used in the tertiary treatment. The iron chloride is pumped into the aeration tank and reacts with the liquid orthophosphates to form solid iron-phosphate complexes, which can then be removed in the sludge. As a result of this process, the levels of Zn from this plant are slightly higher in the sludge compared with those from Longford or Tullamore as there would have been additional zinc in the iron chloride. Table 1 shows the concentrations of heavy metals in soil above which, sewage sludge cannot be applied to agricultural land [20]. Where the pH of the soil is consistently higher than 7, the values set may be exceeded by not more than 50%, provided that there is no resulting hazard to human health, the environment, or groundwater. We have shown that with the exception of nickel the majority of the individual determinations of the other metals tested for in the three plants were below the maximum allowable value where available but the values observed sometimes exceeded the values for use in subsequent agricultural application. The efficiencies of the plants in this regard need to be re-evaluated.

The nondetection of chromium in sewage influent, effluent, and river water was noteworthy given the levels detected in the corresponding matrices of river sediment and sewage sludge. Plant type *Y* had higher levels of zinc as attributed to the tertiary flocculation process. 

## 4. Conclusion

The testing for endocrine disrupting metals was carried out on rivers, landfills, and on sewage treatment plants in the border midlands and western region of the Republic of Ireland. The results should inform debate about the relevance of environmental quality standards in terms of added risk contribution from background metal levels. The results, for the most part, Cd excepted, were below intervention values or maximum allowable concentrations, with however the most notable result being that of lead in leachate sediment from location number 6. As expected, landfill leachate and sewage effluents were identified as sources of entry of these metals into the environment. Ongoing monitoring of sludge metal contents is also indicated.

## Figures and Tables

**Table 1:** Oestrogenicity of metals (data amalgamated from Choe, Martin, and EPA).

Compound/metal	Relative potency	Limit (*μ*g/L) for drinking water (EPA Ireland) S.I. No. 278/2007	Limit (*μ*g/L) for hard surface water (EPA Ireland). Water quality dangerous substances S.I. No. 12/2001	Maximum value for conc. of metals in sludge for use in agriculture (mg/kg) S.I. No. 267/2001	Limit of detection (ng/L) of the AAS method	Sources
Oestradiol	1	No data	N/A	No data	N/A	An endogenous natural hormone.
Ni	1	20	50	300	2.4	Catalysts, by-product of industrial processes.
Mn	0.91	50	N/A	No data	0.4	Power plants, coke ovens.
Cd	0.74	5	N/A	20	0.8	Tobacco, burning coal, plating effluent.
Zn	0.66	3000 (abstraction level)	100	2500	0.8	Dyes, wood preservative, alloys.
Co	0.29	No data	N/A	No data	2.0	Paint drier, colour pigment, car/aeroplane exhausts.
Pb	0.27	10	10	750	5.6	Mining, ore processing, smelting, refining, recycling, and disposal.
Cr	0.27	50	30	No data	2.0	Tanneries, by-product from steel manufacture or anodising plants.
Hg	0.27	1	N/A	16	N/A	Metal smelters, batteries, cement manufacture.
Cu	0.2	2000	30	1000	0.4	Plumbing, smelting operations.

**Table 2:** Average values for master variables over a six-month period for the river sediments and leachate sediment, 2004-2005.

Location	County	Site no.	Mean pH (RSD 0.3%)	Mean % carbonate (RSD 0.6%)	Mean % organic content
*River*					
Mote Park (River Hind)	Roscommon	1	7.66	19.99	5.66
Banagher (R. Shannon)	Offaly	2	8.12	23.23	4.02
Shannon Harbour (Grand Canal)	Offaly	3	8.04	22.66	2.66
Athlone Lock (R. Shannon)	Westmeath	4	7.83	23.97	3.15
Lanesborough (R. Shannon)	Longford	5	7.89	24.49	3.69

*Leachate*					
Burgess Park (R. Shannon)	Westmeath	6	7.75	22.55	6.20
Ballydonagh (R. Al)	Westmeath	7	8.18	23.84	4.08
Derryclure (Tullamore River)	Offaly	8	7.28	24.40	8.13

**Table 3:** Metal content in river sediment in the Irish midlands region (mg/kg_dw_) over a six-month period, 2004-2005.

					Sediment		Concentration		(mg/kg_dw_)		

Metal	Target^†^ value (mg/kg)	Intervention^†^ value (mg/kg)	Site no.	Overall average result*	Nov	Dec	Jan	Feb	Mar	Apr	Mean % RSD
Ni	35.0	100.0	1	<TV, <IV	22	25	29	27	21	46	2.6
			2	<TV, <IV	36	55	28	29	26	15	1.9
			3	<TV, <IV	25	32	26	33	31	20	4.1
			4	<TV, <IV	70	27	4	26	27	19	4.3

Mn	U	U	1	—	402	334	81	113	198	505	4.1
			2	—	495	1050	130	64	26	271	3.4
			3	—	246	244	271	102	102	241	4.5
			4	—	484	112	86	90	89	207	6.9

Cd	0.8	12.0	1	>TV, >IV	<LOD	9	14	20	30	20	9.8
			2	>TV, >IV	<LOD	15	12	26	32	15	11.2
			3	>TV, >IV	1	13	19	19	25	18	9.6
			4	>TV, >IV	16	16	14	28	29	18	7.8

Zn	140.0	720.0	1	<TV, <IV	82	74	117	56	118	277	0.5
			2	<TV, <IV	59	101	74	55	156	132	0.6
			3	<TV, <IV	110	132	149	170	179	59	0.6
			4	<TV, <IV	81	170	45	132	161	144	0.3

Co§	9.0	33.0	1	>TV, <IV	19	18	15	11	17	16	5.9
			2	>TV, <IV	19	26	21	18	19	10	5.2
			3	>TV, <IV	2	29	18	18	16	12	6.7
			4	>TV, <IV	48	22	7	16	16	11	7.1

Pb	85.0	530.0	1	>TV, <IV	141	220	210	140	235	170	1.1
			2	>TV, <IV	141	223	228	145	255	185	1.3
			3	>TV, <IV	188	350	358	150	415	285	1.6
			4	>TV, <IV	296	350	140	128	495	330	1.3

Cr	100.0	380.0	1	<TV, <IV	45	39	61	46	39	10	9.6
			2	<TV, <IV	49	59	115	35	34	9	7.4
			3	<TV, <IV	40	42	54	35	42	10	8.4
			4	<TV, <IV	124	42	12	27	28	10	10.8

Cu	36.0	190.0	1	<TV, <IV	23	14	36	39	11	29	1.2
			2	<TV, <IV	14	22	6	9	7	8	2.3
			3	>TV, <IV	100	81	17	36	29	20	1.1
			4	<TV, <IV	15	20	1	6	98	16	4.3

U: unknown. *Note*: Site no. 5 has been omitted as sediment was only procured for a single month due to a consistently high water level and high currents. *<TV is below the target value, >TV is above the target value, and <IV is below the intervention value, whilst >IV is above the intervention value.

^†^Amalgamated Dutch values for soils and sediments and Danish values for soils [3, 4, 23].

**Table 4:** Metal content in leachate sediment in the Irish Midlands Region (mg/kg_dw_) over a six-month period, 2004-2005.

Metal	Site no.	Sediment concentration (mg/kg)						

		Nov	Dec	Jan	Feb	Mar	Apr	Grand mean % RSD (each run performed in triplicate)
Ni	6	31	31	30	23	23	21	3.3
	7	25	26	26	32	32	23	4.1

Mn	6	229	179	98	31	40	158	1.9
	7	50	755	815	404	104	332	2.7

Cd	6	2	13	21	25	30	15	6.3
	7	<1	17	24	27	33	17	9.1

Zn	6	81	230	147	139	209	191	0.5
	7	87	143	94	78	190	123	0.5

Co	6	27	22	18	16	16	10	3.5
	7	6	21	21	16	20	12	4.1

Pb	6	3525	1375	1375	1225	1475	550	2.3
	7	146	260	242	140	268	220	2.4

Cr	6	125	44	26	24	31	11	5.8
	7	48	44	38	24	41	10	7.1

Cu	6	50	40	22	26	30	24	1.6
	7	13	19	11	7	29	12	2.1

**Table 5:** Mercury concentrations in river sediments and leachate sediments, 2004-2005.

Location	Sample month	Concentration mg/kg_dw_	Comment on result
River	Nov	0.02	<TV, <IV
Hind	Jan	0.01	<TV, <IV
Sediment	Mar	<0.05	<TV, <IV

Grand Canal/	Dec	0.01	<TV, <IV
Shannon	Jan	0.12	<TV, <IV
Harbour	Feb	0.09	<TV, <IV
Sediment	Apr	0.02	<TV, <IV

River Al/	Dec	<0.05	<TV, <IV
Ballydonagh	Feb	<0.05	<TV, <IV
Sediment	Apr	<0.05	<TV, <IV

River Shannon/	Nov	0.12	<TV, <IV
Burgess Park	Dec	0.05	<TV, <IV
Sediment	Feb	0.10	<TV, <IV
	Apr	0.05	<TV, <IV

Derryclure landfill sediment/silt	Apr	0.19	<TV, <IV

**Table 6:** Field results of master variables for river water samples for the month of April.

Parameter	Site no.1	Site no. 2	Site no. 3	Site no. 4	Site no. 5
Dissolved oxygen (mg/L)	10.1	10.5	10.9	11.1	10.2
pH	7.35	7.94	8.01	8.05	7.83
Temperature (°C)	9.8	8.7	9.2	8.0	9.6
Total solids (g/L)	0.3882	0.2019	0.3561	0.2271	0.2041
Suspended solids (g/L)	0.3840	0.0009	0.0041	0.0031	0.0021
Dissolved solids (g/L)	0.0042	0.2010	0.3520	0.2240	0.2020

**Table 7:** Concentrations in river samples *μ*g/L – April.

Site no.	Cu	Zn	Ni	Mn	Pb
1	23	602	11	44	55
2	16	85	2	18	39
3	15	299	6	29	40
4	151	592	7	29	48
5	28	264	9	63	45

*Note*: Co, Cr, and Cd were not detected in liquid samples. Limits of detection are as per Table 1. RSD ranges for these analyses were from 0.4% to 2.1%.

**Table 8:** Field results of master variables for leachate sample locations, 2005.

Parameter	Burgess Park (Site no. 6)	Derryclure SW1	Derryclure N3	Derryclure ST2	Ballydonagh groundwater	Ballydonagh surface water (site no. 7)
Dissolved Oxygen (mg/L)	11.7	4.6	4.7	3.2	3.1	11.1
pH	8.12	7.36	7.36	7.47	7.36	7.58
Temperature (°C)	7.6	8.9	9.0	8.6	5.1	7.9
Total Solids (g/L)	0.3186	1.637	1.091	0.856	0.3458	0.4041
Suspended Solids (g/L)	0.0036	0.112	0.075	0.495	0.0008	0.0051
Dissolved Solids (g/L)	0.315	1.525	1.016	0.361	0.345	0.399

*Note*: SW1: surface water from sampling point 1; N3: sampling location at noise point 3; ST2: sampling point at silt trap 2.

**Table 9:** Concentrations in landfill leachate *μ*g/L – April.

Location/site no.	Cu	Cr	Zn	Ni	Mn	Pb
6	34	<LOD	207	8	14	45
Ballydonagh groundwater	24	0.10	130	8	144	46
7	21	<LOD	113	10	114	29
Derryclure SW1	9	7	174	8	623	55
Derryclure N3	24	<LOD	149	5	495	56
Derryclure ST2	26	<LOD	936	10	235	56

*Note*: LOD Cr 0.002 *μ*g/L.

RSD ranges for these analyses were from 0.1% to 5.0%.

**Table 10:** Concentrations of metals found in sludge (mg/kg) for each treatment type.

Metal	Site type	Sediment concentration (mg/kg)							

Δ (MPC mg/kg)		Nov	Dec	Jan	Feb	Mar	Apr	Kruskal-Wallis *P* value for *X* versus *Y* versus *Z* ^†^	Mean % RSD
Ni 300–400	*X*	2905	2310	2210	2278	2180	375	.220	1.5
	*Y*	3625	4700	4055	3408	910	325		4.1
	*Z*	2633	2925	3208	3010	2480	175		2.1

Mn	*X*	243	358	185	658	698	410	.109	3.5
	*Y*	320	220	150	740	240	1642		1.9
	*Z*	178	180	123	90	241	615		2.4

Cd 20–40	*X*	15	16	12	15	20	100	.059	11.3
	*Y*	7	8	13	9	6	20		14.6
	*Z*	15	14	15	13	17	30		15.2

Zn 2500–4000	*X*	1187	1048	952	925	981	2001	.484	0.4
	*Y*	3400	1625	1002	1845	643	1745		0.3
	*Z*	846	844	888	1162	1081	2826		0.4

Co	*X*	8	10	8	9	10	7	.044	2.3
	*Y*	23	13	20	15	10	10		7.5
	*Z*	16	19	24	37	9	5		5.6

Pb 750–1200	*X*	150	300	325	250	375	750	.109	1.3
	*Y*	150	100	250	275	150	700		0.5
	*Z*	300	350	325	375	375	675		2.0

Cr n/a	*X*	253	296	410	372	473	372	.003	8.2
	*Y*	213	35	134	212	68	137		7.8
	*Z*	253	334	403	486	455	289		9.1

Cu 1000–1750	*X*	212	200	155	145	155	703	.164	3.4
	*Y*	625	373	373	478	33	1003		1.7
	*Z*	275	240	265	435	398	531		2.0

*Hg 16–25	*X*	—	0.16	—	0.34	—	0.42	.054*	—
	*Y*	—	0.42	—	0.36	—	0.40		—
	*Z*	—	0.88	—	1.14	—	0.88		—

Δ Maximum permitted concentration as per EU directive 278/86.

^†^Using Minitab15. This test, applied following evidence of nonnormality of the observed levels, tests for equality of medians of the treatment types (*X*, *Y*, and *Z*), that is, if *P* < .05 we conclude that the medians are not the same. Adjustments made for ties as required.

*Only 3 samples from each site were chosen for mercury analysis

**Table 11:** Concentrations (*μ*g/L) of metals in sewage influent and effluent.

Site type	Cu	Cr	Zn	Ni	Mn	Pb
*X* In	22	<LOD	231	10	45	19
*X* Out	16	<LOD	128	4	102	14
*Y* In	18	<LOD	1297	15	410	8
*Y* Out	26	<LOD	1484	5	47	20
*Z* In	28	<LOD	228	9	288	33
*Z* Out	20	<LOD	96	4	53	13

*Note*: LOD Cr 0.002 *μ*g/L.
